# The Atlanta Medical and Surgical Journal

**Published:** 1887-10

**Authors:** 


### THE ATLANTA MEDICAL AND SURGICAL JOURNAL.

The death of Dr. James A. Gray, the managing editor of this JOURNAL, is a heavy blow. THE JOURNAL will be continued, however, as heretofore. Its new management will do everything to keep it up to the high standard it attained under the able leadership of the lamented Dr. Gray.

